# Postoperative recurrence and systemic metastasis of large cell neuroendocrine carcinoma of the breast: a case report including T-DXd treatment

**DOI:** 10.3389/fonc.2026.1747084

**Published:** 2026-02-13

**Authors:** Siying Zhu, Yinxi Qu, Dong Chen, Xiaomao Luo

**Affiliations:** Department of Ultrasonography, Yunnan Cancer Hospital, The Third Affiliated Hospital of Kunming Medical University, Kunming, Yunnan, China

**Keywords:** breast cancer, large cell neuroendocrine carcinoma, postoperative recurrence, systemic metastasis, trastuzumab deruxtecan (T-DXd)

## Abstract

We report a rare case of large-cell neuroendocrine carcinoma (LCNEC) of the breast in a 49-year-old woman. The patient presented after discovering a right breast mass five years earlier. Mammography revealed a high-density mass and enlarged axillary lymph nodes. Biopsy confirmed invasive carcinoma with neuroendocrine differentiation. Initially, she received TE chemotherapy, followed by carboplatin and etoposide (CBP) due to disease progression. After eight cycles, she underwent modified radical mastectomy and postoperative radiotherapy. One year later, the patient developed metastases in the liver and bones, with a liver biopsy confirming recurrent LCNEC. Given the aggressive nature of the disease, trastuzumab deruxtecan (T-DXd) was initiated on October 11, 2025. Follow-up imaging demonstrated significant reduction in hepatic and peritoneal metastases, with partial response (PR) as the best observed response. Treatment with T-DXd is ongoing, highlighting its efficacy in managing aggressive LCNEC with distant metastasis.

## Introduction

Primary neuroendocrine carcinoma of the breast (NECB) is an extremely rare subtype of breast cancer, representing approximately 0.3%–0.5% of all breast malignancies (1). In the 2019 World Health Organization (WHO) 5th Edition of the Classification of Tumors of the Breast, primary neuroendocrine neoplasms (NENs) of the breast were defined as rare and poorly characterized entities, categorized as: (i) well-differentiated neuroendocrine tumors (NETs, corresponding to grade 1–2 carcinoid-like tumors) and (ii) poorly differentiated neuroendocrine carcinomas (NECs), including small-cell and large-cell subtypes (2).

Among these, large-cell neuroendocrine carcinoma (LCNEC) is exceptionally rare, with only a small number of published cases. Early clinical diagnosis can be difficult because imaging and histopathological characteristics may mimic those of conventional invasive breast carcinoma (3). No standardized treatment strategy has been established, and LCNEC is frequently associated with high aggressiveness, rapid progression, and early distant metastasis, resulting in a poor prognosis (4).

Here, we present a rare case of breast LCNEC. Despite receiving standardized comprehensive management—including chemotherapy, radical surgery, and radiotherapy—and despite achieving negative surgical margins, the patient developed systemic recurrence within one year. By reviewing the clinical course, pathological features, and subsequent treatment decisions, we aim to shed light on the biological aggressiveness and potential therapeutic considerations for this uncommon tumor subtype.

## Case report

**Figure 1:**
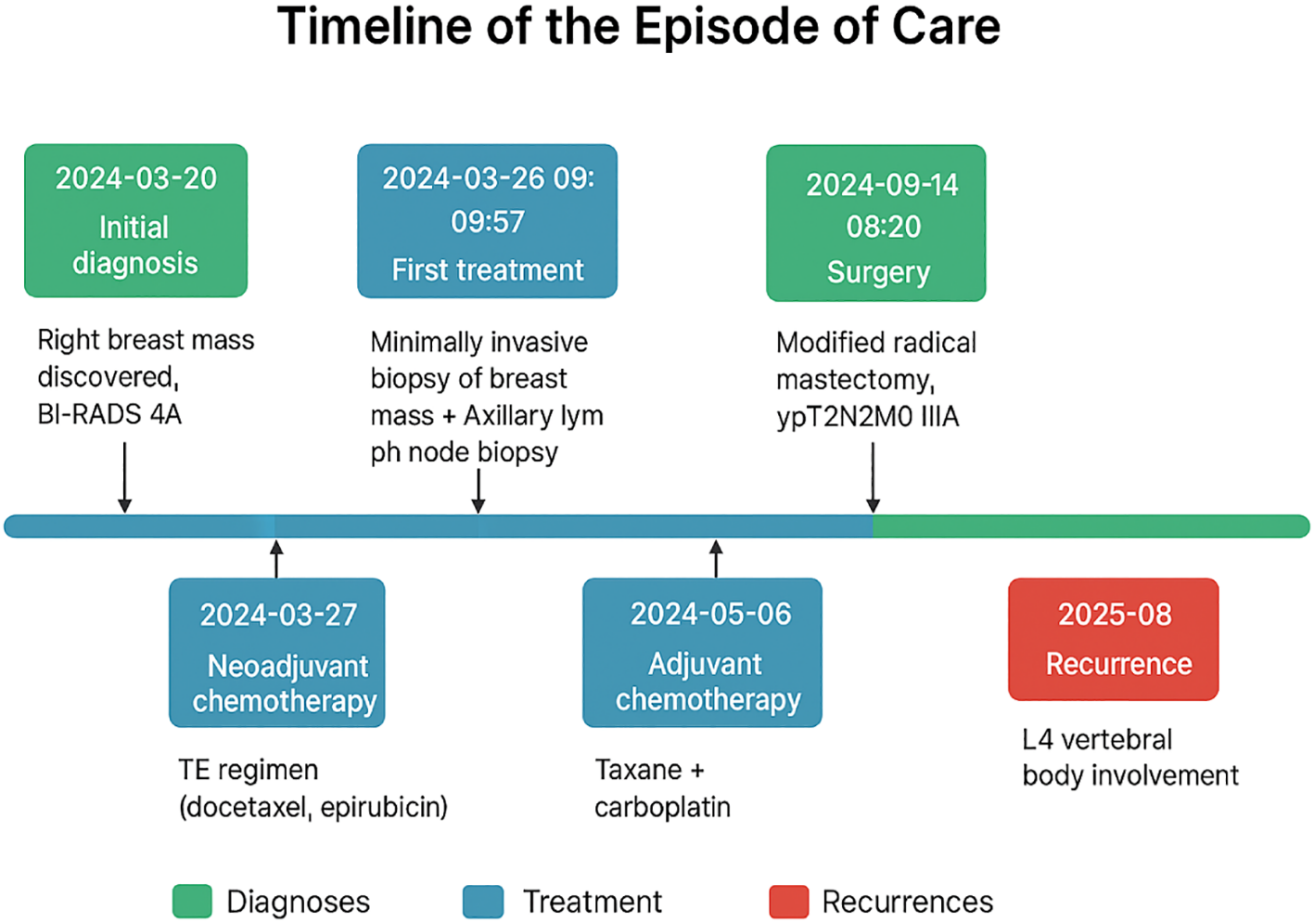
Time-line.

**Figure 2:**
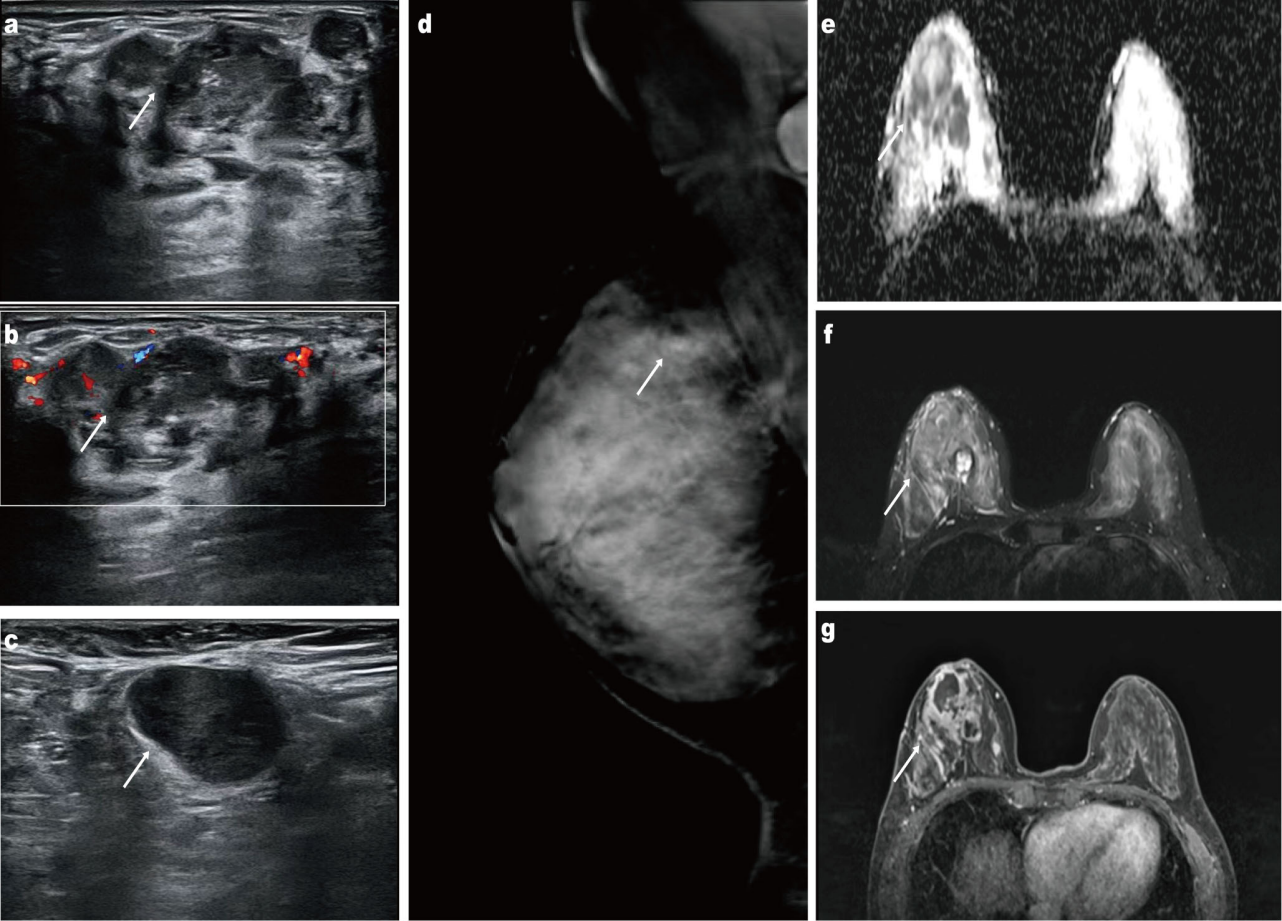
Ultrasonography of the right breast reveals an irregular hypoechoic mass with indistinct margins **(a)**. Color Doppler ultrasonography shows internal vascular flow within the lesion **(b)**. Ultrasonography of the right axilla demonstrates an enlarged lymph node with cortical thickening **(c)**. Maximum-intensity-projection breast MRI image displays a lobulated enhancing mass in the right breast **(d)**. Apparent diffusion coefficient (ADC) map shows markedly restricted diffusion in the lesion **(e)**. Axial contrast-enhanced T1-weighted MRI demonstrates heterogeneous enhancement of the mass **(f)**. Axial T2-weighted MRI reveals high-signal intensity within the lesion **(g)**.

A 49-year-old woman presented to our hospital one year ago with progressive enlargement of a right breast mass that had been detected five years earlier, for which she had not received any specific treatment (**Figure 1**). The patient had no family history of malignancy, hereditary conditions, or infectious diseases. Mammography showed a high-density irregular mass in the upper quadrant of the right breast with ipsilateral axillary lymphadenopathy. Ultrasonography revealed multiple irregular hypoechoic masses with poorly defined margins in the outer, upper outer, and upper quadrants of the right breast. MRI identified a lesion measuring approximately 4.6 cm × 3.0 cm × 3.5 cm in the upper quadrant, exhibiting non–mass-like enhancement with clustered ring enhancement (**Figure 2**).

**Figure 3:**
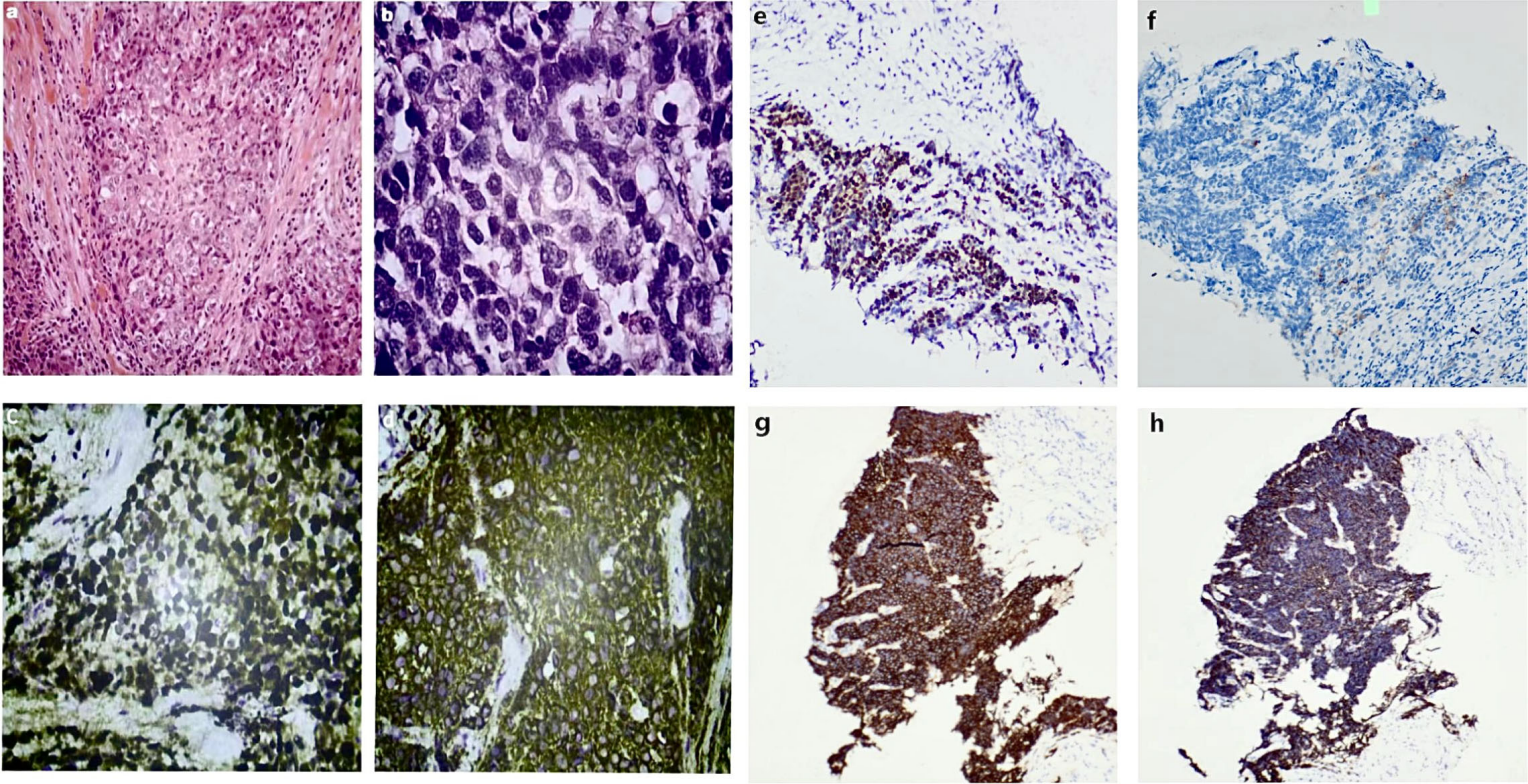
Hematoxylin–eosin staining of the breast core biopsy shows invasive ductal carcinoma **(a)** and, at higher magnification (×20), irregular tumor nests with marked nuclear atypia **(b)**. Immunohistochemical staining of the breast specimen demonstrates diffuse cytoplasmic positivity for synaptophysin (×200) **(c)** and a high Ki-67 proliferation index of approximately 80% (×200) **(d)**, supporting invasive carcinoma with neuroendocrine differentiation. Immunohistochemical analysis of the liver biopsy specimen shows strong positive expression of chromogranin A (CgA) **(e)**, CD56 **(g)**, and synaptophysin **(h)** (×200), PD-1 immunostaining shows positive tumor-infiltrating lymphocytes, while tumor cells are negative **(f)** (×200),confirming metastatic neuroendocrine carcinoma.

Breast and axillary needle biopsies were performed. Histopathological examination confirmed invasive carcinoma with focal neuroendocrine differentiation. Immunohistochemical analysis demonstrated low expression of ER (2%) and PR (5%), HER2 score 2+ with negative FISH amplification, AR positivity in approximately 50% of tumor cells, a high Ki-67 proliferation index (80%), diffuse cytoplasmic positivity for synaptophysin, partial positivity for CK5/6, and preserved GATA3 expression. Based on the combined HE and immunohistochemical findings, the lesion was diagnosed as invasive carcinoma with neuroendocrine differentiation (**Figure 3**).

The diagnosis was challenging because classical neuroendocrine morphology was not prominent in the initial biopsy specimen, and the tumor showed mixed features overlapping with invasive ductal carcinoma. Differential diagnoses included invasive ductal carcinoma with neuroendocrine differentiation, primary breast neuroendocrine carcinoma, and metastatic neuroendocrine neoplasm from an extramammary origin. The diffuse synaptophysin expression, together with GATA3 positivity and clinical exclusion of a non-breast primary site, supported a breast origin. The high Ki-67 index (80%) and focal neuroendocrine differentiation suggested an aggressive biological behavior. Therefore, the final diagnosis of invasive carcinoma with neuroendocrine differentiation of breast origin was established.

The patient began the first cycle of TE chemotherapy on March 28, 2024, with TXT 120mg IV infusion and EPI 120mg IV infusion. Subsequent cycles were administered every 21 days, completing three cycles by May 8, 2024. Following disease progression, the regimen was switched to P(White)Cbp on May 28, 2024, also administered every 21 days. After two cycles, treatment was further changed to albumin-bound paclitaxel (400mg IV) and carboplatin (0.6g IV), with two additional cycles completed by August 22, 2024. After eight cycles, a modified radical mastectomy of the right breast was performed. The intraoperative surgical margins were each greater than 2 cm from the tumor edge. Both frozen-section analysis and postoperative pathology confirmed that the superior, inferior, medial, and lateral skin margins were free of cancer. Postoperative pathology demonstrated extensive tumor necrosis, vacuolated cancer cells, nuclear enlargement and atypia, and pronounced stromal fibrosis. In addition, tumor cells showed marked nuclear pleomorphism with occasional bizarre nuclear forms. The tumor stroma exhibited prominent fibrosis accompanied by infiltration of lymphocytes, multinucleated giant cells, and histiocytic reaction. These morphological features, together with the immunohistochemical findings, supported the diagnosis of poorly differentiated invasive carcinoma with neuroendocrine differentiation. The pathological stage was ypT2N2M0, stage IIIA. Radiotherapy was administered thereafter.

**Figure 4:**
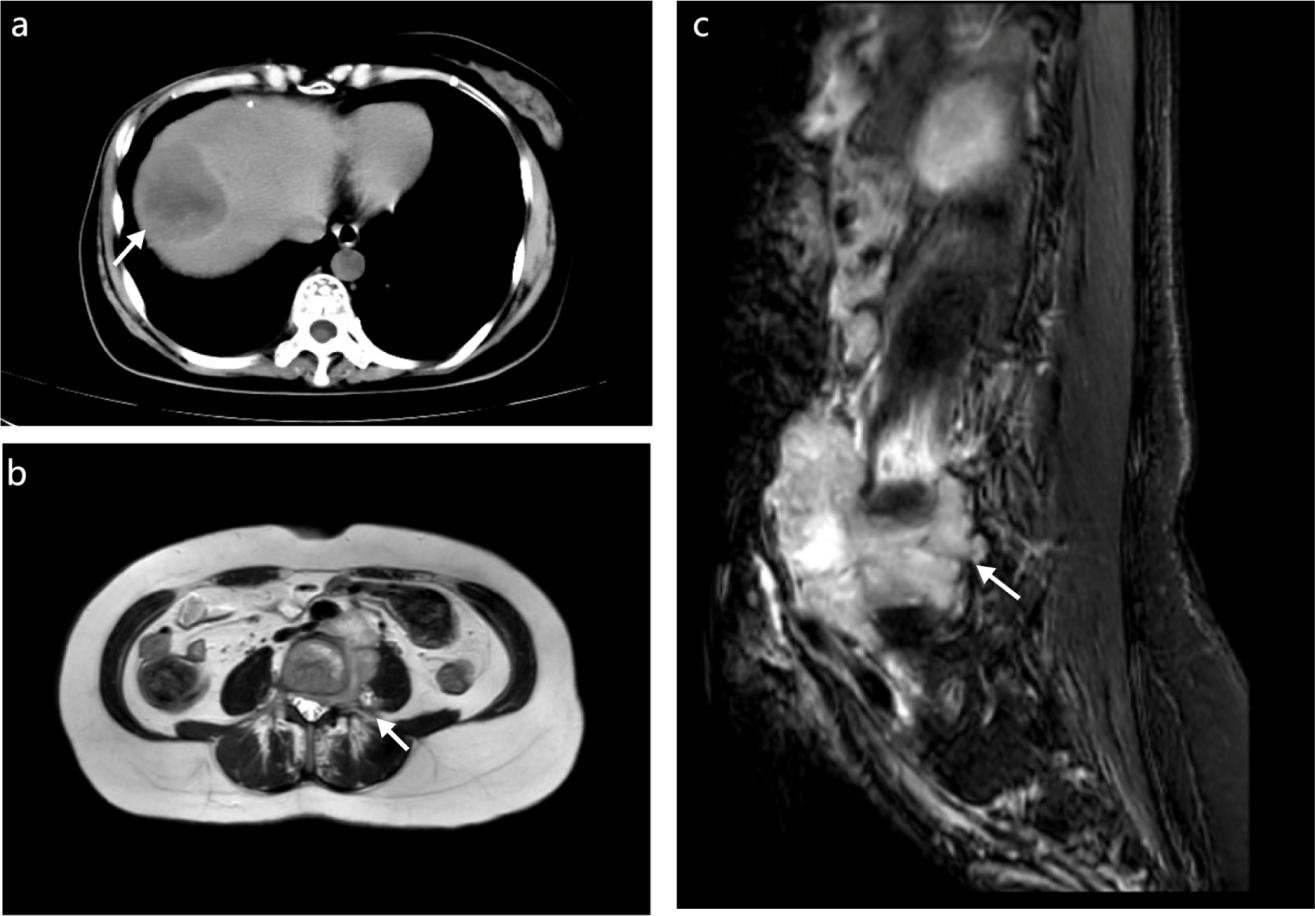
Non-contrast computed tomography of the upper abdomen shows a hypodense lesion in the right hepatic lobe, consistent with hepatic metastasis **(a)**. Axial magnetic resonance imaging demonstrates a pelvic mass involving the adnexal region and vertebral metastasis **(b)**. Sagittal T2-weighted MRI of the lumbar spine reveals an osteolytic lesion involving the vertebral body, indicating bone metastasis **(c)**.

According to standard treatment protocols, the disease would be expected to stabilize. However, one year after surgery, the patient developed marked lumbar and back pain. Lumbar MRI showed bone destruction involving the L4 vertebral body and left paravertebral region, with a soft tissue mass extending into the intervertebral foramen and spinal canal, compressing the adjacent dural sac. Abdominal ultrasound and CT identified abnormal echogenic areas in the right hepatic lobe and perivascular regions of major abdominal vessels; altered bone density in the C7 transverse process, L4, and S1 vertebral bodies; and right pleural thickening with nodules, all suggestive of metastasis (**Figure 4**).

A subsequent liver biopsy confirmed metastatic neuroendocrine carcinoma. Immunohistochemical analysis demonstrated negativity for ER and HER2, moderate to strong PR expression in approximately 80% of tumor cells, a high Ki-67 proliferation index (80%), loss of broad-spectrum cytokeratin expression, weak CK7 positivity, and diffuse positivity for CD56, synaptophysin, and chromogranin A. PD-1 expression was observed in tumor-infiltrating lymphocytes, whereas tumor cells were negative (**Figure 3**). Based on the patient’s clinical history, the diagnosis was confirmed as recurrent metastatic large-cell neuroendocrine carcinoma of the breast. Notably, despite negative primary tumor margins and standardized comprehensive treatment, the patient developed widespread recurrence within a short interval, indicating highly invasive behavior and substantial metastatic potential.

As the metastatic lesions were confirmed to be of breast origin and considering the tumor’s pathological features, treatment with trastuzumab deruxtecan (T-DXd) was initiated on October 11, 2025, at a dose of 300 mg IV every 3 weeks. Subsequent cycles were administered on November 6 and December 1, 2025. Beginning with the December 1, 2025 cycle, following multidisciplinary evaluation and detailed physician–patient discussion, toripalimab (240 mg IV) was added, and combination therapy was continued.

The decision to administer trastuzumab deruxtecan in combination with toripalimab was made outside the context of a clinical trial. Given the absence of established treatment guidelines for recurrent metastatic breast large-cell neuroendocrine carcinoma and the aggressive disease course after standard therapies, this approach was considered an individualized therapeutic attempt driven by clinical necessity. The treatment plan was discussed in a multidisciplinary team meeting involving medical oncologists, radiologists, and pathologists, where the potential risks and anticipated benefits were carefully evaluated. After detailed counseling regarding the experimental nature of the combination, possible toxicities, and the uncertainty of clinical benefit, the patient agreed to proceed, and the physician–patient discussion as well as the patient’s request for the addition of immunotherapy were documented in the medical record. Follow-up imaging at the most recent evaluation demonstrated a marked reduction in the size of both hepatic and peritoneal metastatic lesions compared with prior examinations, indicating a partial response (PR) as the best observed objective response to T-DXd–based therapy. Treatment with the same regimen is ongoing.

At the time of reporting, the progression-free interval from initiation of T-DXd therapy is approximately 3.5 months, and progression-free survival has not yet been reached. The overall systemic treatment strategy has been dynamically adjusted according to disease status, without a predefined treatment end point.

Regarding safety, the patient tolerated T-DXd well. Based on clinical assessment, laboratory testing, cardiac monitoring, and serial imaging, no evidence of interstitial lung disease (ILD) or other severe T-DXd–related toxicities has been observed to date.

During T-DXd–based therapy, the patient was generally able to tolerate the treatment and did not report a clear decline in her daily activities. In routine follow-up visits, no severe treatment-related symptoms that markedly affected her quality of life were identified. She experienced some anxiety related to her disease and ongoing therapy, but this was manageable. The treatment goals and potential risks were explained to her, and she expressed understanding and a willingness to continue therapy.

## Discussion

Neuroendocrine carcinoma of the breast (NECB) is an extremely rare malignancy, comprising approximately 0.3%–0.5% of all breast cancers (1). The 2019 WHO classification designates it as a breast neuroendocrine neoplasm (BNEN) and divides poorly differentiated forms into small-cell and large-cell subtypes (2). The large-cell subtype is even less common, with only a few case reports available in the literature.

Diagnosis requires careful histopathological evaluation of biopsy specimens. Classical neuroendocrine morphological features may be absent, and mixed growth patterns involving invasive ductal and lobular carcinoma are often observed (5). Immunohistochemistry typically demonstrates positivity for neuroendocrine markers such as synaptophysin, chromogranin A, and CD56 (2, 6), in addition to breast-associated markers such as GATA3 or CK7. In this case, the immune profile—with diffuse synaptophysin expression, partial CK7 positivity, and GATA3 positivity—supported a breast origin. Metastatic neuroendocrine carcinoma from an extra-mammary primary site was excluded based on negative systemic imaging findings and an immunohistochemical profile consistent with breast origin, although an occult primary site cannot be completely ruled out.

Because of the rarity of NECB, no universally accepted treatment guideline exists. Documented objective responses are primarily from hormone receptor-positive cases treated with endocrine therapy (7, 8) and from small-cell NECB treated with platinum-based chemotherapy (9). Previous reports indicate higher local recurrence and distant metastasis rates, as well as poorer survival, in NECB compared with conventional invasive ductal carcinoma (IDC, NOS) (4, 10). Some authors have noted that poorly differentiated large-cell NEC may be less responsive to standard breast cancer chemotherapy regimens—including taxanes, anthracyclines, and platinum agents—suggesting more aggressive biological behavior (11).

Despite receiving chemotherapy, modified radical mastectomy, and postoperative radiotherapy with negative surgical margins, the patient developed multiple liver, bone, and pleural metastases within one year, indicating a strong systemic invasive potential of this tumor. In addition, the postoperative pathological stage of ypN2 reflects extensive residual nodal disease after neoadjuvant therapy, which is a well-recognized adverse prognostic factor associated with significantly reduced disease-free and overall survival (12). Therefore, the early systemic recurrence observed in this case is likely attributable to both the aggressive biological behavior of LCNEC and the high-risk ypN2 nodal status, rather than failure of local control alone. This clinical course may also be related to the high proliferation index (Ki-67, 80%) and potential molecular alterations such as TP53, RB1, or PI3K pathway abnormalities, which warrant further investigation. Although genomic testing was not performed in this case, these molecular drivers represent critical areas for future investigation to better understand the aggressive nature of LCNEC.

Changes in hormone receptor expression between primary and metastatic breast cancer have been widely reported. Several studies have demonstrated discordance in ER, PR, and HER2 status between primary tumors and metastatic lesions, which may significantly influence therapeutic decision-making and prognosis (13, 14). In our case, the primary tumor showed low ER and PR expression, whereas the metastatic liver lesion demonstrated complete loss of ER expression with markedly increased PR positivity, supporting the concept of receptor status conversion during tumor progression. This phenomenon may reflect clonal selection under treatment pressure or tumor evolution and highlights the importance of re-biopsy of metastatic lesions to guide personalized therapy.

Although the patient showed limited response to conventional chemotherapy, emerging treatment options such as antibody–drug conjugates (ADCs) and immunotherapy may offer additional benefit. After the diagnosis of liver metastasis, the patient received T-DXd (trastuzumab deruxtecan), with good tolerance and improvement in clinical status. Recent randomized clinical trials have demonstrated that targeting low levels of HER2 with trastuzumab deruxtecan is a superior therapeutic approach compared with untargeted chemotherapy in patients with HER2-low metastatic breast cancer. In these studies, trastuzumab deruxtecan showed a significant improvement in progression-free survival compared with standard chemotherapy in patients with hormone receptor–positive, HER2-low metastatic breast cancer, as well as in populations including HER2-low and HER2-ultralow tumors. No new safety signals were observed (15, 16).

Although our patient was classified as HER2-negative by conventional criteria, the aggressive disease course, lack of response to standard chemotherapy, and emerging evidence supporting the activity of T-DXd in HER2-low or ultralow settings provided a reasonable clinical basis for its use. Nevertheless, the application of T-DXd in breast large-cell neuroendocrine carcinoma remains exploratory and should be interpreted cautiously, pending further prospective validation.

The rationale for considering the addition of immunotherapy to trastuzumab deruxtecan in this case was hypothesis-driven and supported by emerging translational evidence suggesting potential synergy between antibody–drug conjugates and immune checkpoint blockade. Beyond direct cytotoxicity, trastuzumab deruxtecan has been reported to induce immunogenic cell death, which may promote the release of damage-associated molecular patterns and enhance immune cell infiltration within the tumor microenvironment. In addition, early-phase clinical studies, including DESTINY-Breast08, are actively evaluating combinations of antibody–drug conjugates with immune checkpoint inhibitors in HER2-low breast cancer, indicating that such strategies are under clinical investigation. Although these data do not establish a standard of care, they provide biological plausibility for exploring this combination in selected clinical scenarios where therapeutic options are limited. In the future, targeted personalized approaches—including HER2-low ADCs, PARP inhibitors, or combinations of PD-1/PD-L1 inhibitors—may provide new therapeutic strategies.

In summary, breast LCNEC is a rare and highly aggressive tumor subtype characterized by early recurrence, distant metastasis, and resistance to standard chemotherapy. This case demonstrates that early systemic recurrence may occur even when surgical margins are negative and comprehensive treatment has been completed, reflecting the tumor’s inherently aggressive biology. Early identification, molecular profiling, and development of more effective targeted and immunotherapy-based regimens are essential directions for future research.

This case has several strengths, including detailed pathological confirmation, documentation of early systemic recurrence, receptor status conversion, and response to antibody–drug conjugate therapy. However, several limitations should be acknowledged. First, this is a single-case report, and the findings cannot be generalized to all patients with breast LCNEC. Second, no genomic testing or comprehensive molecular profiling was performed in this instance, which limits our ability to confirm the specific molecular alterations driving this patient’s aggressive disease course. We acknowledge this as a significant limitation, and future studies incorporating multi-omics data are essential to identify potential therapeutic targets for this rare malignancy.

Despite these limitations, this case contributes valuable clinical and pathological information to the limited literature on breast LCNEC and highlights the need for further multicenter studies.

## Data Availability

The original contributions presented in the study are included in the article/supplementary material. Further inquiries can be directed to the corresponding author.
